# Automated detection of neonatal pulmonary hypertension in echocardiograms with a deep learning model

**DOI:** 10.1038/s41390-025-04404-3

**Published:** 2025-09-24

**Authors:** Holger Michel, Ece Ozkan, Kieran Chin-Cheong, Anna Badura, Verena Lehnerer, Stephan Gerling, Julia E. Vogt, Sven Wellmann

**Affiliations:** 1 University of Regensburg Faculty of Medicine, University Children’s Hospital Regensburg (KUNO), Hospital St. Hedwig of the Order of St. John, Regensburg, Germany; 2 Department of Computer Science, ETH Zürich, Zürich, Switzerland

## Abstract

**Background:**

In infants, pulmonary hypertension (PH) increases morbidity and mortality. Echocardiography, though standard, is time- and expertise-demanding. We propose a deep learning approach for automated PH detection using standard echocardiography videos, validated by the systolic eccentricity index (EIs).

**Methods:**

The training and validation set comprised 975 videos and the held-out set 378 videos, including five echocardiographic standard views from infants aged 3–90 days, taken between 2018–2021 and 2021–2022, respectively. Echocardiograms were labeled as PH (EIs < 0.82) and healthy (EIs ≥ 0.87). After preprocessing and random segmentation of all videos into 13.530 frames, spatial and spatio-temporal convolutional neural network architectures were used for training of a PH prediction model and gradient-weighted class activation mapping for explainability.

**Results:**

The best single-view performance was achieved using parasternal short axis view (AUROC spatial and spatio-temporal: 0.91 and 0.94 in validation set, 0.93 and 0.88 in held-out set, respectively). Combination of three standard views improved accuracy with AUROC 0.96 and 0.90 in validation (spatio-temporal) and held-out set (spatial), respectively. Saliency maps revealed model focus on clinically relevant regions, including interventricular septum and left atrial filling.

**Conclusions:**

The presented deep learning model for automated detection of PH in neonates shows high accuracy, explainability, and reproducibility.

**Impact:**

- This study presents a deep learning model that enables accurate, automated detection of pulmonary hypertension in infants using standard echocardiography videos, enhanced and evaluated with eccentricity index, an established and prognostically relevant echocardiographic parameter.
- The parasternal short-axis view showed the best single-view performance, combined views further improved accuracy.
- Explainability through saliency maps supports clinical acceptance, highlighting physiologically relevant regions in the decision process.
- It adds novel evidence to the literature, demonstrating the utility of spatio-temporal convolutional neural networks for early, non-invasive diagnosis.
- The model provides a scalable and reproducible tool for routine PH screening, potentially improving early detection and outcomes.

## Introduction

Pulmonary hypertension (PH) in neonates, infants and children contributes significantly to both morbidity and mortality, arising from a wide spectrum of cardiac, pulmonary, and systemic diseases.^1^ Persistent pulmonary hypertension of the newborn (PPHN) is typically associated with acute respiratory failure and can be a life-threatening complication. In preterm infants, bronchopulmonary dysplasia (BPD) is a major etiologic factor for frequently developing PH. Similarly, several other developmental lung diseases like congenital diaphragmatic hernia and pediatric heart disease may be associated with PH.^1,2^ Therefore, early detection and continuous monitoring of PH are essential for optimizing clinical management, underscoring the need for accurate and reliable diagnostic tools.

Echocardiography is the first-line diagnostic modality for PH due to its non-invasive nature and accessibility. Different echocardiographic parameters and indices have been introduced to diagnose and quantify PH using different modalities like M-Mode, Doppler- and Tissue-doppler echocardiography.^3^ As pulmonary arterial pressure increases, right ventricular pressure rises relative to the left ventricle, leading to alterations in cardiac geometry. These include systolic flattening of the interventricular septum, which can be visualized using 2D echocardiography. One quantitative measure of this geometric change is the left ventricular systolic eccentricity index (EIs), which has been validated as a useful parameter in evaluating PH in neonates and infants.^4–6^ EIs has demonstrated strong correlations with invasive hemodynamic measurements in pediatric PH.^7^ Furthermore, increased EIs has been shown to be diagnostic for PPHN and a predictor for death in these neonates.^8^ In preterm infants an elevated EIs was also associated with unfavorable outcome like death and BPD related PH.^9^

Despite its clinical utility, echocardiographic assessment of PH remains time consuming and expertise demanding. A reliable automated machine-learning approach for the diagnosis of pulmonary hypertension could reduce workload and increase diagnostic confidence in the clinical setting. Few papers have addressed automatic detection of PH using echocardiography in the adult population. While Leha et al.^10^ proposed an approach that is based on manually extracted echocardiographic parameters, the method of Zhang et al.^11^ does not require manual feature extraction. The latter method uses a single four-chamber view (A4C) and works with static echocardiography frames exploiting spatial patterns only. Similarly, Diller et al.^12^ used deep learning to estimate PH based on static echocardiographic images, and proposed a model trained to segment the cardiac chambers and extract geometric information throughout the cardiac cycle. However, these models generally lack interpretability and explainability, and importantly, none have been validated in pediatric populations, including neonates and infants—groups in whom PH presents unique diagnostic challenges and imaging characteristics.

In our previous work, we pioneered a deep learning-based model capable of predicting PH in newborns and infants using a novel multi-view approach with echocardiographic standard view loops.^13^ In this methodical groundwork, the model was trained and evaluated using annotations derived from expert visual assessments of the videos by a pediatric cardiologist.

Building on this methodological breakthrough, the current study introduces a key innovation: the integration of the left ventricular systolic eccentricity index (EIs) into the model architecture. EIs is a clinically validated, prognostically meaningful marker that correlates with disease severity and outcomes in pediatric PH. By incorporating this quantitative and interpretable parameter, we aim to enhance both the diagnostic accuracy and clinical relevance of our model. This represents a critical step toward developing an explainable, automated tool for early and reliable PH detection in neonates, infants, and children—a domain that remains largely unexplored in existing AI-driven cardiology research.

## Methods

### Study design and dataset

We conducted a single center retrospective study approved by the local ethic committee Regensburg (20-2052_1-104). Echocardiographic examinations (ECHOs) of neonates, infants and children were extracted and analyzed retrospectively. We included ECHOs in the study that were performed by the same senior pediatric cardiologist and ultrasound device between 2018 and 2022. From each examination, 2D echocardiography videos were extracted in up to five standard planes. These five standard planes included an apical four-chamber view (A4C), a parasternal long-axis view (PLAX), and three parasternal short-axis views; a midventricular view between the level of the papillary muscles and the level of apical part of the mitral valve (PSAX-P), at the level of semilunar valves (PSAX-S), and an apical short-axis view (PSAX-A). Only ECHOs with 2D videos in the parasternal short axis view at the midventricular level (PSAX-P) and at least one additional standard plane were included in this study.

The dataset was collected in two batches. The first batch, the training and validation set contained 199 ECHOs performed between 2018 and 2021. It was used for training of our model and internal validation within a cross-validation framework as described below. These ECHOs comprised a total of 975 standard videos, which consisted of 199 PSAX-P, 191 PSAX-A, 193 PSAX-S, 194 A4C and 198 PLAX. The second batch was independent from the first and served as held-out test set. It contained 77 ECHOs performed between 2021 and 2022. These ECHOs comprised a total of 378 standard videos, which consisted of 77 PSAX-P, 75 PSAX-A, 75 PSAX-S, 77 A4C, and 74 PLAX.

All ECHOs were performed using a GE Logic S8 ultrasound machine with the S4–10 transducer at 6 MHz frequency operating on 25 frames per second. The average video length was 163 ± 68 frames, with each ECHO comprising around 10 to 15 heartbeats. The average spatial size of the original 2D videos was 1440 ×866 pixels. All data was pseudonymized.

### Labeling of the echocardiograms

For each ECHO, we calculated the systolic eccentricity index (EIs) which is the ratio of the systolic left ventricular diameter perpendicular to the interventricular septum divided by the diameter parallel to the interventricular septum leading to a decrease in EIs with increasing PH. Of note, some authors in the literature also calculate the EIs as an inverse value (ratio of systolic left ventricular diameter parallel to the ventricular septum divided by the perpendicular diameter), which leads to an increase in EIs with increasing PH. In this study, we used the calculation described above and all values given are based on this ratio. The EIs was measured in the PSAX-P plane in end systole (final frame of LV free wall contraction) measuring the diameters from compact myocardium.^3,6,7^ For the training and validation set, one measurement was carried out for each ECHO, for the test set the EIs was measured in two different heartbeats and the average of these was used for the ECHO annotations.

Burkett et al.^7^ related EIs measurements to invasive hemodynamics and found an EIs of ≤ 0.86 to best identify the presence of PH. Nagiub et al.^3^ recommended a cut-off of 0.81 for diagnosis of PH in their systematic review. In line with these recommended cut-off values for the EIs, in this study, we used < 0.82 to label patients with PH and ≥ 0.87 for healthy patients. The ECHOs with EIs between 0.82 and 0.87 were marked as low-confidence and dropped from the study. In addition, the corresponding additional views were also labeled for each ECHO.

### Preprocessing and data augmentation

**Fig. 1:**
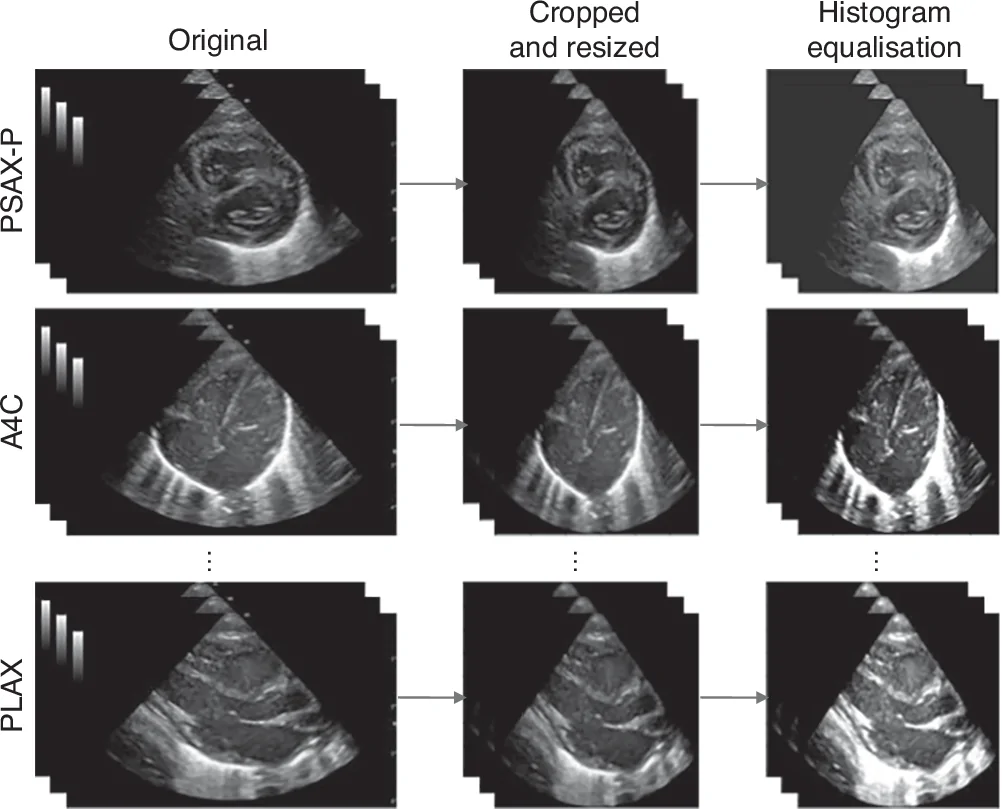
Dataprocessing of the echocardiographic videos including cropping and resizing the videos in the first step followed by histogram equalization. PSAX-P parasternal short axis view, A4C apical four chamber view, PLAX parasternal long axis view.

Preprocessing and data augmentation was performed in the following way: We cropped and masked the 2D echocardiography videos to eliminate information (such as the graphical user interface of the US device, additional text or signals) outside the scanning sector and resized them to 224 × 224 px^2^ using bilinear interpolation. We then applied histogram equalization to distribute the pixel intensities to the full range of gray-scale values and normalized them. The proposed procedure is shown in Fig. 1.

During training, we applied two types of image augmentations to increase the robustness of the model and eliminate spurious correlations that might be present in the data. We used both intensity and spatial transformations. The former ensured that the learned model is invariant to image intensity variations. The latter, on the other hand, increased resilience against different zoom settings of the US machine, the actual size of the heart, and/or the transducer placements. In particular, we applied the following random transformations to each sequence: sharpness and brightness adjustment, gamma correction, the addition of salt and pepper or Gaussian noise, variation of the background with different amounts of speckle noise, rotation up to 15°, width and height shifts up to 10%, scaling down to 80%, zooming up to 120%.

### Training for prediction of pulmonary hypertension in infants

**Fig. 2:**
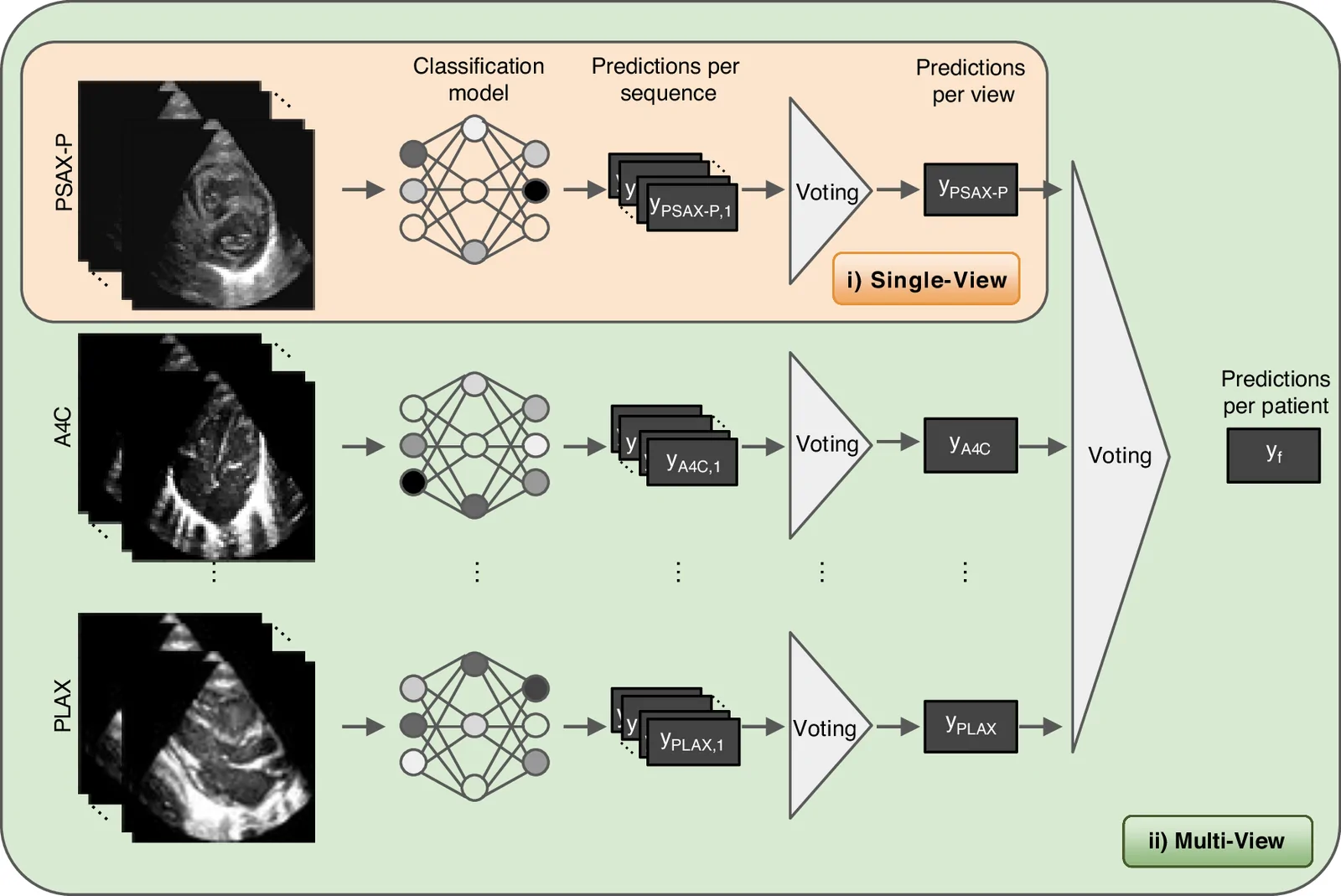
Overview of the automated PH assessment based on spatio-temporal patterns of the echocardiographic videos using majority voting. Panel (**i**) displays a single view and (**ii**) a multi-view approach trained on e.g., MV-3 using the three views (PSAX-P, parasternal short axis view; A4C, apical four chamber view; PLAX, parasternal long axis view).

Figure 2 provides an overview of the methodology of training for the predictions. As mentioned above, for each ECHO, 2D echo videos were available in multiple standard planes showing the heart from different views contributing to the prediction at the ECHO-level.

### Spatial and Spatio-temporal approach

We employed either a 3D-CNN architecture with residual connections and spatio-temporal convolutions across frames or a 2D-CNN architecture with spatial convolutions for each frame.^13,14^ While using the 2D-CNN architecture, the learned model integrated only spatial information in the learning process, with the 3D-CNN architecture, temporal information was integrated into the learning process additionally. The learned model integrated spatial as well as temporal information into the learning process. Hereafter, the architecture with 3D-CNN will be called spatio-temporal method and 2D-CNN spatial method.

#### Single-view

We first processed each view separately (Fig. 2 (i)). We extracted n = 10 shorter video sequences from each echo-video, covering one heartbeat on average. For the 3D-CNN, we randomly selected one frame as the starting frame, followed by k − 1 consecutive frames, with a total of k = 12 frames. For the 2D-CNN, we set k = 1. We then aggregated sequence-level predictions {y_view,i_}_i=1,…,n_ through majority voting, i.e., by selecting the most frequently predicted label, to a view-level prediction y_view_. The view-level confidence was then defined as C=|y_view_^*^|/n, where|y_view_| was the count of the most frequently predicted label from the list of predictions for the n sequences (or frames for 2D-CNN) of a given ECHO per view.

#### Multi-view

We employed a multi-view approach to further increase the method’s robustness by combining the models trained on each available view (Fig. 2 (ii)). The final subject-level prediction, y_f_, was then achieved by majority voting of the view-level predictions. In the case of a tie, the prediction of the model(s) with higher confidence was selected. We tried different approaches for view aggregation, but simple majority voting performed the best.

### Interpretability of pulmonary hypertension prediction in infants

**Fig. 3:**
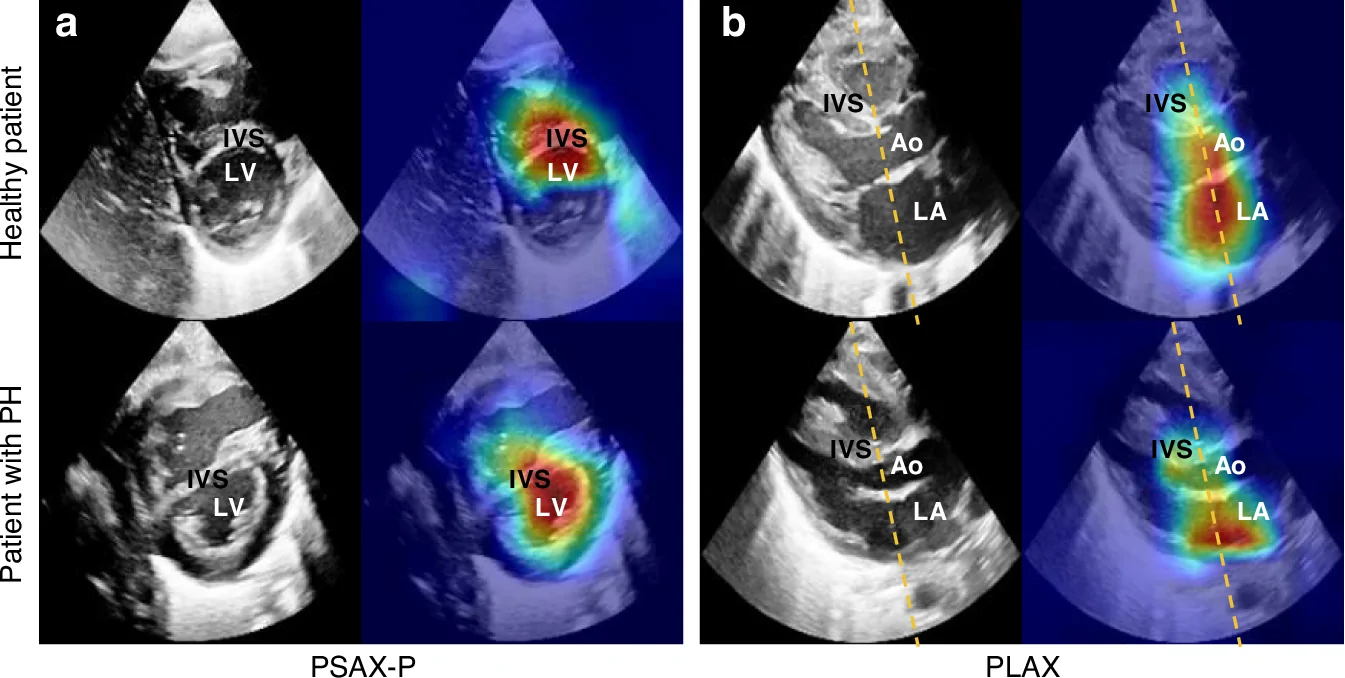
ECHO frames of healthy and patient with PH (left), as well as the IP-PHN saliency maps (right). Panel (**a**) shows the PSAX-P, parasternal short axis  view, and (**b**) the PLAX, parasternal long axis view. The yellow line shows how the M-mode for the LA: Ao measurement is extracted. The highlighted pixels feature crucial cardiac structures. LV left ventricle, IVS interventricular septum, LA left atrium, AO aortic root.

We complemented our predictions with saliency maps from each view to increase accountability and clinical usability of our proposed method’s (Fig. 3). In this study we used Gradient-weighted Class Activation Mapping (Grad-CAM) as saliency method, an explanation method that exploits the gradients of any target concept flowing into a given convolutional layer to produce a coarse localization map highlighting the critical regions in the image for predicting the label.^15^ Grad-CAM was originally proposed for 2D-CNNs. Therefore, we extended Grad-CAM to 3D-CNNs that process spatio-temporal video inputs as described before.^13^

### Implementation details

Our method was developed using the Python programming language with the PyTorch deep learning library. Our experiments were run on a cluster containing different NVIDIA GeForce graphic cards: GTX 1080, GTX 1080 Ti, RTX 2080 Ti with 2048 MB RAM per processor core. For both of the architectures, we used a ResNet-18 as the model backbone.^16^

To deal with class imbalance, we employed a weighted random sampler, which samples elements from the dataset using their inverse class weight as their sample weight, ensuring that for every epoch, the model sees an approximately equal number of samples from each class. Additionally, to avoid potential overfitting on the dataset, throughout training, we applied on-the-fly data augmentation and we continuously augmented each sample during training with a probability of 90%.

Each model was trained for around 150 epochs per view, minimizing the (categorical) cross-entropy loss with the Adam^17^ optimizer. The learning rate and weight decay were set to 0.001, while the batch size was set to 8 video sequences for the spatio-temporal and 64 for the spatial method.

### Experimental setup

For training of the model, the training and validation split of the ECHOs was used. We performed a stratified 10-fold cross-validation, such that the data was randomly split ten times into 20% validation set and 80% training set. Note that the splitting into training and validation sets was done on a patient basis. We then evaluated the trained model on the held-out test set.

As classification metrics, we evaluated the area under the receiver operating characteristic (AUROC, one-vs-one), frequency-weighted F1-score, weighted precision, weighted recall and balanced accuracy as commonly used metrics. The multi-view AUROC was computed from the output probabilities of the most confident model selected by the majority voting. Results were averaged over the folds, and the mean and standard deviation were reported on the patient level.

## Results

The baseline data of the patients at the time of echocardiography for the training and validation set and the held-out test set is given in Table 1. The median gestational age at birth was 31 + 6 weeks and 28 + 5 weeks, the median age 8 days and 13 days and the median weight 2750 g and 1600 g, respectively.

**Table 1:** Baseline characteristics and number of ECHOs and echocardiographic standard views for the training and validation set as well as the held-out set

	Training and validation set		Held-out set	
Gestational age at birth, median (IQR)	31 + 6 (26 + 2 - 39 + 3)		28 + 5 (26 + 5 - 30 + 1)	
Age in days at scan, median (IQR)	8 (3–71)		13 (3–57)	
Weight in gram at scan, median (IQR)	2750 (2250–3750)		1600 (1320–2700)	
	Healthy group	PH group	Healthy group	PH group
Gestational age at birth, median (IQR)	30 + 3 (26 + 3–38 + 4)	38 + 3 (26 + 0–40 + 0)	27 + 6 (26 + 2–29 + 6)	38 + 3 (34 + 6–39 + 3)
Age in days at scan, median (IQR)	3 (1–79)	15 (4–71)	21 (5–58)	1 (0–3)
Birthweight in gram, median (IQR)	2750 (1750–3750)	3150 (2750–3360)	1400 (1205–1930)	3274 (2750–3628)
Echocardiograms in total, n	199		77	
-healthy group, n (%)	116 (58%)		57 (74%)	
-PH-group, n (%)	69 (34%)		18 (23%)	
-Term / Preterm, n (%)	44 (63%) / 25 (33%)		12 (66%) / 6 (33%)	
-excluded, n (%)	14 (7%)		2 (3%)	
Standard view videos in total, n	975		378	
Breakdown of performed views:				
PSAX-P, PSAX-A,	199, 191,		77, 75,	
PSAX-S, A4C, PLAX	193, 194, 198		75, 77, 74	

*PSAX-P* midventricular parasternal short-axis view, *PSAX-S* at the level of semilunar valves, *PSAX-A* apical short-axis view, *A4C* apical four-chamber view, *PLAX* parasternal long-axis view, *IQR* interquartile range, *GA* gestationl age, Term, GA ≥ 37 + 0; Preterm, GA < 37 + 0; n, number

**Fig. 4:**
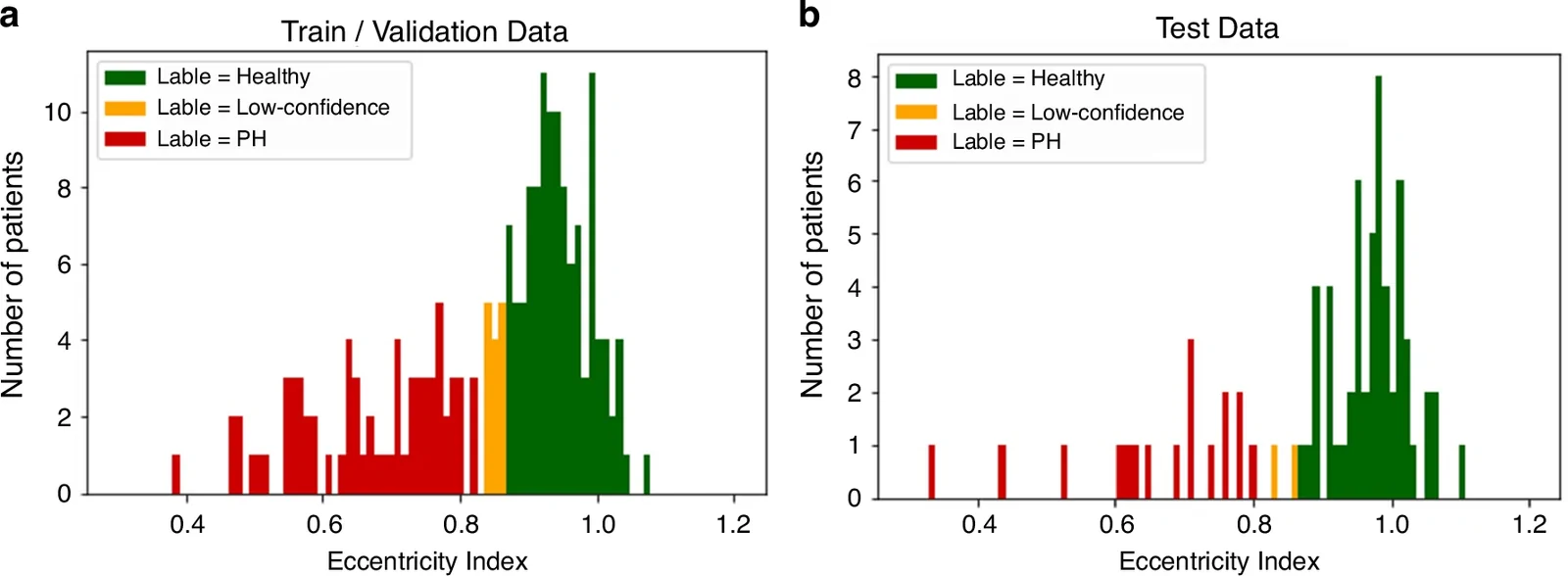
Histogram of the dataset showing the distribution of the left ventricular systolic eccentricity index and the corresponding labeling of the ECHOs. Panel (**a**) includes 199 newborns in the training and validation split and (**b**) containing 77 patients in the held-out test split.

EIs was calculated for each ECHO. In the training and validation set 14 patients were excluded due to an EIs between 0.82 and 0.87 (low confidence). 116 (58%) ECHOs were labeled as Healthy (EIs ≥ 0.87) and 69 (34%) ECHOs were labeled as PH (EIs ≤ 0.82). Figure 4 demonstrates the distribution of the EIs in the dataset. The final training and validation set contained 185 ECHOs. In the held-out test set two patients were excluded due to an EIs between 0.82 and 0.87 (low confidence). 57 (74%) ECHOs were labeled as Healthy (EIs ≥ 0.87) and 18 (23%) ECHOs were labeled as PH (EIs ≤ 0.82) (Fig. 4). The final held-out test set contained 75 ECHOs.

The clinical conditions resulting in elevated right ventricular pressure were classified based on criteria published on the 7th World Symposium on Pulmonary Hypertension.^18^ In 13 ECHOs the increased right ventricular pressure was due to congenital heart disease (group 1.4.4), in 51 ECHOs to PPHN (group 1.6) and in 23 ECHOs to developmental lung disease (group 3.7).

We first performed an empirical evaluation of the model on the training and validation split of the dataset as described above. Table 2 shows the quantitative results of the five single views (A4C, PLAX, PSAX-P, PSAX-S, PSAX-A) (see Fig. 2 (i)), as well as the multi-view approach (see Fig. 2 (ii)) obtained by majority voting. In particular, we combined the three views A4C, PLAX and PSAX-P for MV-3 and all views for five different views for MV-All.

**Table 2:** Results from spatio-temporal (a) and spatial (b) approaches for PH diagnosis in neonates and infants on validation data

	View	AUROC	F1-Score	Precision	Recall	Balanced Accuracy
(a)	A4C	0.90 ± 0.04	0.88 ± 0.03	0.89 ± 0.03	0.88 ± 0.03	0.88 ± 0.03
	PLAX	0.91 ± 0.03	0.89 ± 0.03	0.90 ± 0.03	0.89 ± 0.03	0.89 ± 0.03
	PSAX-P	0.94 ± 0.03	0.92 ± 0.03	0.93 ± 0.03	0.92 ± 0.03	0.92 ± 0.03
	PSAX-S	0.83 ± 0.06	0.83 ± 0.04	0.84 ± 0.04	0.83 ± 0.04	0.82 ± 0.03
	PSAX-A	0.89 ± 0.06	0.88 ± 0.05	0.88 ± 0.05	0.87 ± 0.05	0.88 ± 0.05
	**MV-3**	**0.96** ± **0.02**	**0.95** ± **0.03**	**0.95** ± **0.02**	**0.95** ± **0.02**	**0.94** ± **0.03**
	MV-All	0.96 ± 0.02	0.94 ± 0.03	0.94 ± 0.03	0.94 ± 0.03	0.94 ± 0.03
(b)	A4C	0.92 ± 0.03	0.89 ± 0.03	0.89 ± 0.03	0.89 ± 0.03	0.88 ± 0.04
	PLAX	0.94 ± 0.03	0.91 ± 0.04	0.92 ± 0.04	0.91 ± 0.04	0.92 ± 0.04
	PSAX-P	0.91 ± 0.06	0.89 ± 0.06	0.90 ± 0.05	0.89 ± 0.06	0.89 ± 0.05
	PSAX-S	0.90 ± 0.06	0.88 ± 0.05	0.89 ± 0.04	0.88 ± 0.05	0.88 ± 0.05
	PSAX-A	0.89 ± 0.05	0.86 ± 0.06	0.87 ± 0.06	0.86 ± 0.06	0.87 ± 0.06
	MV-3	0.95 ± 0.03	0.93 ± 0.03	0.94 ± 0.03	0.93 ± 0.03	0.93 ± 0.03
	MV-All	0.96 ± 0.03	0.94 ± 0.04	0.95 ± 0.04	0.94 ± 0.04	0.94 ± 0.04

*MV-3* refers to the majority voting of A4C, PLAX, and PSAX-P views. *MV-All* refers to majority voting of all five views. The best results for each task have been highlighted in **bold**.

Using the single-views, PSAX-P showed the best performance for PH prediction using the spatio-temporal method and achieved an AUROC of 0.94, F1-score of 0.92 and Balanced Accuracy of 0.92, followed by PLAX (Table 2 (a)). We observed that both PSAX-P and PLAX were relatively confident in their prediction, with an average confidence of correct predictions of 0.92 and 0.91, respectively, while it decreases to 0.83 and 0.84 for wrong predictions. Since the apical four-chamber view (A4C) is one of the most commonly used views for cardiovascular disease diagnosis, we also evaluated A4C and our evaluation showed that it was similarly discriminative as PSAX-P and PLAX, with an AUROC of 0.90, F1-score of 0.88 and Balanced Accuracy of 0.88, which were slightly lower than the other two views.

We used majority voting to combine the results from the different views in order to increase the robustness and accuracy of the proposed approach. For MV-3 (combined results from PSAX-P, PLAX, A4C) the AUROC increased from 0.94 to 0.96, the F1-score improved from 0.92 to 0.95, the Balanced Accuracy from 0.92 to 0.94 with a relative increase ratio of 2.1%, 3.2% and 2.2%, respectively. When the other two short-axis views were furthermore included (MV-All), we got an AUROC of 0.96 an F1-score and a Balanced Accuracy of 0.94.

As described above we trained and evaluated our model also in the spatial approach with a 2D-CNN architecture. The results are shown in Table 2(b). Overall the performance of our model using the spatial approach was comparable to the spatio-temporal approach with the best accuracy when combining all views in MV-All.

In the second step, we evaluated our trained model on the held-out test set. Overall, the method demonstrated high accuracy for the PH detection on the unseen dataset. The results are presented in Table 3. We again performed a spatio-temporal and a spatial approach which achieved comparable scores. The best performance on the held-out test data for a single view was achieved using PSAX-P in the spatial approach, with an AUROC of 0.93, F1-score of 0.86 and Balanced Accuracy of 0.82. The combination of all views (MV-All) showed a slight further improvement with an AUROC from of 0.90, F1-score of 0.89 and Balanced Accuracy of 0.84. Our results demonstrate that the proposed spatial and spatio-temporal approach for PH detection achieved high accuracy, particularly when combining multiple echocardiographic views. The majority voting method improved predictive performance, highlighting the benefit of integrating different perspectives. These findings suggest that our approach could enhance automated PH detection and support clinical decision-making in neonatal and pediatric populations.

**Table 3:** Results from spatio-temporal (a) and spatial (b) approaches for PH diagnosis in newborns on held-out test data

	View	AUROC	F1-Score	Precision	Recall	Balanced Accuracy
(a)	A4C	0.83 ± 0.05	0.79 ± 0.04	0.80 ± 0.04	0.79 ± 0.05	0.72 ± 0.06
	PLAX	0.83 ± 0.03	0.86 ± 0.04	0.86 ± 0.04	0.86 ± 0.04	0.76 ± 0.05
	PSAX-P	0.88 ± 0.05	0.80 ± 0.05	0.81 ± 0.04	0.80 ± 0.05	0.73 ± 0.07
	PSAX-S	0.81 ± 0.07	0.75 ± 0.05	0.78 ± 0.04	0.73 ± 0.06	0.70 ± 0.05
	PSAX-A	0.85 ± 0.04	0.81 ± 0.04	0.82 ± 0.04	0.81 ± 0.04	0.74 ± 0.06
	MV-3	0.88 ± 0.07	0.87 ± 0.04	0.88 ± 0.03	0.88 ± 0.03	0.80 ± 0.08
	MV-All	0.87 ± 0.07	0.88 ± 0.02	0.88 ± 0.02	0.88 ± 0.02	0.82 ± 0.04
(b)	A4C	0.81 ± 0.04	0.80 ± 0.03	0.80 ± 0.03	0.81 ± 0.03	0.71 ± 0.04
	PLAX	0.83 ± 0.04	0.84 ± 0.02	0.86 ± 0.03	0.86 ± 0.02	0.73 ± 0.06
	PSAX-P	0.93 ± 0.04	0.86 ± 0.04	0.87 ± 0.05	0.86 ± 0.04	0.82 ± 0.07
	PSAX-S	0.87 ± 0.03	0.79 ± 0.04	0.81 ± 0.03	0.79 ± 0.04	0.74 ± 0.04
	PSAX-A	0.87 ± 0.04	0.81 ± 0.05	0.84 ± 0.03	0.81 ± 0.06	0.78 ± 0.05
	MV-3	0.90 ± 0.03	0.88 ± 0.02	0.89 ± 0.02	0.89 ± 0.02	0.81 ± 0.04
	**MV-All**	**0.90** ± **0.04**	**0.89** ± **0.02**	**0.90** ± **0.02**	**0.90** ± **0.02**	**0.84** ± **0.04**

*MV-3* refers to the majority voting of A4C, PLAX, and PSAX-P views. *MV-All* refers to majority voting of all five views. The best results for each task have been highlighted in **bold**.

### Explainability

To increase the clinical usability, we applied a post-hoc analysis of the single-view spatio-temporal and spatial convolutions. For each ECHO view, saliency maps highlighted the pixels that were the most relevant for the assessment of PH diagnosis. Figure 3 shows the original ECHO frames of healthy patient and patient with PH (left column) combined with saliency maps using Grad-CAM (right column) corresponding to the significant views.

In the PSAX-P (Fig. 3 (a)), the pixels relevant for classification in our model are located in the region of the interventricular septum. Here, PH leads to a change in the morphology and movement of the intraventricular septum (IVS) and the shape of the left ventricle (LV), which is due to the change in pressure in the right ventricle (RV). In PLAX (Fig. 3 (b)), the saliency maps highlight that besides the IVS the model also focuses on the area around the left atrium (LA) and the aortic root. With increasing PH, the filling of the left atrium may decrease and therefore the relationship between the size of the left atrium and the aortic root may change. Thus, the saliency maps highlight clinically relevant cardiac structures that are important for classification in our model.

## Discussion

Transthoracic echocardiography is a crucial non-invasive modality for the diagnosis of PH, which is a frequent and clinically significant concern in critically ill neonates, infants, and children, given its association with increased morbidity and mortality.^1^ In addition to 2D imaging, modalities such as M-mode, Doppler, and tissue Doppler are routinely employed in screening for PH.^3^ However, comprehensive echocardiographic assessment remains time-intensive and requires specialized expertise. Recent advances in artificial intelligence offer promising opportunities for the automated detection of PH and its underlying causes from cardiovascular imaging. Building upon our prior research, the present study enhances our approach to automated PH detection by leveraging echocardiographic standard view videos. Notably, we integrated the EIs – a well-established echocardiographic parameter with diagnostic and prognostic relevance in neonatal, infant, and pediatric PH. We demonstrated strong model performance, validated through extensive internal stratified cross-validation and evaluation on an independent external test set comprising previously unseen data.

In our previous methodical groundwork we developed a deep learning approach based on 2D standard plane echocardiographic videos of neonates, infants and children.^13^ As a basis for this development, we had used annotations of the videos obtained by visual evaluation of changes in the cardiac geometry like septal flattening. Although this subjective assessment is widely used in clinical practice, the additional use of other more objective parameters in the echocardiographic evaluation of PH is recommended. Therefore in this study we chose to incorporate the EIs into our model to improve performance and make prediction more objectifiable based on evaluated and outcome related criteria. The EIs reflects, objectifies and quantifies the geometrical cardiac changes with increasing PH. It has been recommended in evaluation for PH in neonates and infants^3,5^ and it has been shown to be diagnostic for the presence of PH in different pathophysiological situations in this age group, like preterm infants with BPD^4,6^ neonates with congenital diaphragmatic hernia^19^ and neonates with PPHN.^8^

In preterm infants EIs also correlated well with tricuspid regurgitation jet velocity, another well-established echocardiographic parameter for PH evaluation that is, however, only measurable in a minority of the infants.^4^ Furthermore EIs has been shown to correlate with invasive hemodynamic measurements in pediatric PH.^7^ Studies demonstrated that EIs is a predictor of unfavorable outcome like death in neonates with PPHN^8^ or death and BPD related PH in preterm infants.^9^ Due to the retrospective nature of data acquisition and therefore heterogeneity of the primarily used echocardiographic modalities and measurements, as well as the technical possibilities of standardized retrospective measurement, we decided not to include any further parameters such as Doppler measurements or pulmonary artery structure in this study. An additional integration of such data in future studies may improve accuracy and robustness and provide important data on explainability. As described above, recommended cut-off values for the EIs for diagnosis of PH in neonates, infants and children range between 0.81 and 0.86.^3,7^ For an optimized training of the model according to these values, we excluded ECHOs in the gray zone between 0.82 and 0.87 from the dataset. In this study, therefore, our model was not trained to classify ECHOs in this gray zone. However, in clinical practice, there are patients that fall into this gray zone. Based on the data presented in this study, the performance of the model for prediction in these patients cannot be assessed. Further studies with larger datasets or the use of additional training parameters could further improve the performance of our model in this gray zone and are necessary before clinical use.

In order to make our model as broadly applicable and robust as possible, we conducted the training with infants of different maturity at birth, ages and causes of PH. The most frequent one was PPHN, the most common cause of PH in the neonatal age group. PPHN can be a life-threatening complication that requires immediate treatment, but is usually reversible within the first few days of life with appropriate therapy.^2^ In infants with developmental lung disease in our study, BPD-associated PH was the most common. With increasingly more extremely immature babies surviving, BPD has become a major etiologic factor for PH.^2^ In the smallest group, PH was associated with congenital heart defects. Although the infants in the test dataset differed from the training and validation groups in terms of median maturity at birth, age, and weight (Table 1), our model performed well in automatic PH detection for the ECHOs in the test group, demonstrating robustness even when applied to different patient groups. Due to a lack of data and resulting small subgroups, we did not evaluate the performance of our model separately for different causes of PH. Further studies on even larger data sets may provide more information on this in the future.

In this study, incorporating the eccentricity index (EIs) into model training improved performance over our previously proposed model, both in internal validation and, notably, on the held-out set of previously unseen echocardiograms. In the test set, compared to Ragnarsdottir et al.^13^, the AUROC for the best-performing single view (PSAX-P) improved from 0.90 ± 0.04 to 0.93 ± 0.04 for the spatial approach. Sensitivity improved from 0.80 ± 0.04 to 0.86 ± 0.04 and the balanced accuracy from 0.77 ± 0.04 to 0.82 ± 0.07, respectively. These results highlight the strong performance of our model in detecting PH from previously unseen echocardiograms using PSAX-P as the sole input view.

Our model reached the best performance in the spatial approach when combining all 5 standard planes (MV-All). Again, the classification metrics improved compared to our previously published model with AUROC increasing from 0.86 ± 0.08 to 0.90 ± 0.04, sensitivity from 0.82 ± 0.03 to 0.90 ± 0.02 and balanced accuracy from 0.77 ± 0.04 to 0.84 ± 0.04, respectively.

Similar to Ragnarsdottir et al.^13^ for binary PH detection the performance did not differ when using the spatial compared to the spatio-temporal approach. This indicates that in our model, using the more resource-efficient spatial approach, no deterioration in the binary detection of a PH compared to the spatio-temporal approach has to be accepted. This could be an advantage for applications with limited computing resources, such as on-board use on echo devices. On the other hand, our previous work suggested that there might be advantages of the spatio-temporal approach when applying on the task of PH severity prediction, emphasizing the value of both approaches depending on the task. Future studies have to show if improvement of our model for binary PH detection will also extend to severity prediction.

The improvement in our model’s performance compared to Ragnarsdottir et al. ^13^ may be partly attributed to the exclusion of gray zone examples (EIs between 0.82 and 0.87), which are more ambiguous and difficult to classify with high confidence. These borderline cases likely introduced uncertainty in the previous model, making it harder to distinguish between healthy and PH cases. By removing these low-confidence samples, the model was trained on clearer distinctions between the two classes, leading to improved sensitivity, balanced accuracy, and overall robustness, particularly in previously unseen test data. This refinement highlights the importance of data preprocessing and careful selection of training examples in optimizing model performance.

Apart from the present study and Ragnarsdottir et al.^13^ to the best of our knowledge, no other machine learning models have been published for automated detection of PH in neonates or children using echocardiographic video data. In adult patients, the state-of-the-art method for automated detection of PH using echocardiographic videos was published by Zhang et al.^11^ using the apical four chamber view (A4C) in a spatial approach. No external evaluation with a held out test set was performed in that study. They achieved an AUROC of 0.85 in an internal evaluation. With an AUROC of 0.92 ± 0.03 for the single plane (A4C) and 0.96 ± 0.03 for the multi-view (MV-all) in the internal evaluation our model reached performance metrics well within this range. Diller et al. ^12^ also proposed a deep learning model to predict PH in adults based on static echocardiographic images with very good performance also on a held out test set. None of these adult studies used a spatio-temporal approach which according to our previous results might have advantages when predicting PH severity. Interpretability and explainability methods were also not applied in these adult models.

However, explainability methods can enhance information for clinicians as they highlight structures of potential interest and can help to understand the basis of a prediction. Lack of explainability has been recognized as a limiting factor for wider adoption of machine learning in healthcare.^20^ In our model, saliency maps highlighted cardiac structures as relevant for predictions that reflect typical changes in cardiac geometry with increased right ventricular pressure. For simplicity, the saliency maps of a single frame per patient are shown in Fig. 3. In a clinical setting, the visualizations can be viewed as video containing spatio-temporal or frame-level with spatial explanation.

Several limitations of our method have to be discussed. This study was conducted monocentrically with a single examiner and echo device in order to reduce confounding factors such as different transducers, ultrasound-device settings, video settings like frame rates and resolution for the training and development of the model. The good performance can therefore not be transferred to other settings and further studies are needed to investigate the applicability and validity of the model to echo videos from other examiners and echo devices. Future training of our model with videos more from heterogeneous settings could further increase accuracy and robustness for use in a clinical context. Due to the setting of our study, a large proportion of the included ECHOs were from term and preterm neonates and infants. As shown above, the common pathophysiological backgrounds for PH for this age group were included in our data sets. For older children and also children with other pathophysiological backgrounds for the development of PH, the applicability of the model needs to be evaluated in future studies. Although we chose EIs as an evaluated and recommended parameter for the diagnosis of neonatal or pediatric PH for the training and evaluation of our model, right heart catheterization remains the gold standard for diagnosis and quantification of PH in neonates and children. Future studies with an evaluation of our model with values from invasive PH assessments could further verify the diagnostic accuracy.

Finally, it must be emphasized that the method presented has been evaluated for prediction of echocardiographic findings indicative of right ventricular pressure elevation, but it cannot be used to predict pathophysiological significance or therapeutic relevance as this must be decided in the context of the patient’s history, symptoms, other diagnostic modalities and overall clinical situation.

## Conclusion

In summary, this study presents, to the best of our knowledge, the first explainable automated model for PH assessment based on echocardiographic standard plane videos in neonates and children, incorporating the eccentricity index—a validated, prognostic diagnostic parameter for pediatric PH. The model demonstrated excellent performance in detecting PH with high accuracy, even on previously unseen echocardiograms and using the resource-efficient spatial approach. This makes it a promising tool to support clinicians and enhance diagnostic accuracy. Future studies with larger, more diverse patient cohorts, including different age groups, PH etiologies, and echocardiographic settings, are needed to assess its clinical applicability. Further improvements could be achieved by incorporating additional echocardiographic data, clinical information, and invasive PH assessments.

## Data Availability

The datasets generated during and/or analyzed during the current study are available from the corresponding author on reasonable request.
